# Case Series of Three Neurological Side Effects in Younger-Aged Individuals After Pfizer's mRNA Vaccine

**DOI:** 10.7759/cureus.23779

**Published:** 2022-04-03

**Authors:** Elliot Dinetz

**Affiliations:** 1 Urgent Care/Integrative Medicine, Timeless Health, Miami, USA

**Keywords:** covid-19 neurolo, biontech covid-19 vaccine, covid 19, adverse event, bnt162b2 mrna

## Abstract

With the worldwide goal of ending the pandemic, mRNA vaccines have been introduced as a valuable tool to help achieve both herd immunity and protect the most vulnerable. Neurological side effects from such vaccines have been increasingly documented, but to date, they are still deemed rare with no caution advised per the Centers for Disease Control and Prevention (CDC). As more of the younger population (under 40) are getting vaccinated according to recent approval and CDC recommendations, the real-world safety reporting data on adverse events have yet had time to catch up. We present three distinct neurological events that occurred after the Pfizer mRNA vaccine (BioNTech, Mainz, Germany), without identifiable alternate etiologies, in patients with an average age of 36 years presenting to an urban Florida clinic, all within eight weeks of one another. The presented cases occurred within hours of the second dose and, in one case, after the third booster dose of the Pfizer mRNA vaccine. These cases illustrate rising concerns of risks in widely recognized very low-risk age categories. A clearly delineated risk-benefit strategy likely needs to be implemented.

## Introduction

Since the introduction of mRNA technology vaccines to address the COVID-19 pandemic, the Centers for Disease Control (CDC) maintains that any adverse events are rare, despite concerns. In fact, it has been established that younger individuals and children without serious comorbidities are at very low risk of adverse outcomes post-infection [1]. As far as neurological adverse events following mRNA vaccination, studies show concerningly high odds ratios (2.9) for developing Guillain-Barre syndrome, especially compared to the flu vaccine, for example [2,3]. This was during the height of the pandemic but most obviously in adults without mention of children. This also doesn't account for the potential additive risk of having vaccination and subsequent infection. As transmission despite vaccines is well documented, neurological adverse events are a valid concern in such younger age groups who have their whole lives ahead of them without much understanding of the long-term issues from mRNA technology vaccination. Furthermore, Vaccine Adverse Event Reporting System (VAERS)* *reporting has struggled with underreporting adverse events following vaccines, adding to further concern [4]. 

Nonetheless, we are informed by the CDC that among all age groups, adverse events are rare and often self-limiting after mRNA vaccinations [5]. We see a one-size-fits-all approach that fails to take into account recent data. For example, Moderna (Cambridge, USA) mRNA vaccine showed higher myocarditis incidence in thoseunder 40 years old compared to incidence with infection, while Pfizer (BioNTech, Mainz, Germany) mRNA vaccine have yielded more hemorrhagic stroke in those under 40 years old and higher encephalitis and myasthenic disorders across all ages compared to those with infection [6,7]. Initial authorization studies in low-risk young populations failed to show this and have been underpowered to account for adverse issues. For example, three thousand children were studied for eight weeks after two vaccines were administered in the youngest approved age group (5-11 years old), giving authorization to 50 million children [8]. Here we illustrate three individuals with an average age of 36 who presented with distinct neurological side effects, all within hours after the Pfizer mRNA vaccine. The fact that each presented within eight weeks of one another by January of 2022 to a small volume practice in urban Florida, while none of these individuals had prior vaccination adverse reactions as children, raises serious safety concerns among younger populations. All three patients are still struggling with these symptoms.

## Case presentation

Case 1

A 28-year-old White male Ph.D. student presented to the clinic after dealing with chronic unremitting headaches for six months with no subjective or objective fevers, chills, or rash. Of note, he claims to have a past medical history of apparently being diagnosed with very mild Tourrette syndrome in childhood which he states is "hardly noticeable". He began to experience worsening headaches with physical exertion beginning within four hours after receiving the second dose of the Pfizer COVID-19 vaccine. His pain ranged from a 6/10 to an 8/10 in intensity depending on the amount of exertion, was located between his ears, was non-radiating, and was not associated with a particular time. He maintained the same degree of symptoms as to date without resolution. He did not have symptomatic COVID-19 prior and admitted to contracting COVID-19 two months after completing the two-dose vaccination regimen, but no changes to his symptoms were associated with this. He reported that these headaches limit his ability to exercise; meanwhile, vigorous activity exacerbates symptoms and limits his ability to exercise properly. The patient was referred to neurology for further workup by his primary care physician after a very basic set of labs. The neurologist ordered two different sets of D-dimer's, both unremarkable (see Table 1). The patient was alert and oriented, with no focalized weakness or deficits on a full neurological exam. His cognition, mood, and ambulation were intact as well. Arrhythmia, murmurs, or other cardiovascular findings were not identified. His vitals were also age-appropriate. 

**Table 1:** Laboratory findings for the patient in Case 1

Labs	Values	Reference range
Sodium (Na)	141 mmol/L	135-146 mmol/L
Chloride	106 mmol/L	98-110 mmol/L
Blood urea nitrogen (BUN)	11 mg/dL	7-25 mg/dL
Potassium (K)	5 mmol/L	3.5-5.3 mmol/L
High sensitivity C-reactive protein (hsCRP)	5 mg/L	<1.0 mg/L
Co2	27 mmol/L	20-32 mmol/L
Glucose	80 mg/dL	65-99 mg/dL
Creatinine	0.83 mg/dL	0.60-1.35 mg/dL
Erythrocyte sedimentation rate	6 mm/h	≤ 15 mm/h
D-dimer (repeated twice)	<0.2 mg/L	<1.0 mg/L

Case 2

A 38-year-old white male with no pertinent past medical history, former high school football player and wrestler with no family history, presented to the clinic after having a syncopal episode along with loss of bladder control. The syncopal event lasted around 30 seconds, about two hours after his third Pfizer booster injection. The patient had a recollection of events prior to the event, and his wife, who witnessed this, denied any seizure-like activity or him describing chest pain or arrhythmia. His wife immediately called emergency medical services; EKG and vitals were performed on the scene, which were unremarkable, and the patient was not taken to the hospital. The patient denies palpitations prior to or after the event. The patient was seen by a neurologist where an MRI of the head was ordered with and without contrast which was unremarkable other than a vascular loop anomaly at the level of the left middle cerebral artery bifurcation, which was believed to be unrelated according to his neurology team. Neurological and cardiac exams were normal and unremarkable for any deficits, including cognitive or mood impairment. His labs were also unremarkable, as shown below in Table 2.

**Table 2:** Laboratory findings for the patient in Case 2

Labs	Values	Reference range
Sodium (Na)	140 mmol/L	134-144 mmol/L
Chloride	103 mmol/L	96-106 mmol/L
Blood urea nitrogen (BUN)	12 mg/dL	6-20 mg/dL
Potassium (K)	4.5 mmol/L	3.5-5.2 mmol/L
Co2	22 mmol/L	20-29 mmol/L
Glucose	96 mg/dL	65-99 mg/dL
Creatinine	0.98 mg/dL	0.76-1.27 mg/dL
Calcium	22 mg/dL	8.7-10.2 mg/dL
Vitamin D	20.9 ng/mL	30.0-100.0 ng/ml
Thyroid-stimulating hormone (TSH)	1.2 uIU/mL	0.450-4.500 uIU/mL

Case 3

A 42-year-old nulliparous White female with no past medical history of illness or disease presented to the clinic six months after receiving her two doses of Pfizer COVID-19 vaccine. The patient denies having had COVID-19 previously and describes having developed mild to moderate tinnitus after the first dose. Although she felt it somewhat improving, she was still expected to receive the second shot or compromise her diplomatic status abroad despite her voiced concerns. Her tinnitus worsened after the second dose and has since been persistent. Her physical exam was unremarkable for any auricular or middle ear findings, Dix-Hallpike maneuver, as well as a complete neurological and cardiac exam. The patient was referred by the primary care physician to an otolaryngologist who performed audiometric testing, which was negative, with no subsequent blood work. She was later told that nothing else could be done, and no labs were further ordered.

## Discussion

As we continue to ensure appropriate access to COVID-19 mRNA vaccinations, it is as important to know which groups or individuals may not be such candidates. Neurological issues from vaccination may be the direct toxic effect of S-spike proteins as it damages the blood-brain barrier and can lead to immune dysregulation processes [9]. We also know tinnitus is not an exclusively auditory problem but may result from neurological changes within the auditory system and has been known to have neurological etiology from peripheral and central cytokine-mediated responses [10,11]. Tinnitus is not new in the literature after COVID-19 and mRNA vaccines. In fact, recently, a case series of three patients reported tinnitus following Pfizer mRNA vaccination where two of the three people were also younger reaffirming this as a concerning risk factor [12]. Our case represents a unique scenario since our patient developed tinnitus after the first shot yet became more pronounced and persistent after the second. According to our literature search, this implies causation with a dose-dependent impact not seen before. Since S-spike protein has already been identified as causing blood-brain barrier permeability as well as promoting intestinal permeability, this may explain such an inflammatory phenomenon [13,14].

According to the CDC, headaches have been the neurological adverse reaction most often seen after mRNA vaccination. According to our knowledge, our first case presents a most unusual case of persistent, atypical headache, worse with exertion, never been published before. After two negative D-dimers, neurologists essentially ruled out cerebral venous sinus thrombosis, and no diagnosis nor treatment were given. Given the only history of mild Tourette's syndrome, perhaps underlying neuronal inflammation may have been exacerbated by the vaccine, as we know that headaches, like other neurological issues, are rooted in underlying inflammation [15]. 

Syncope has diverse etiologies ranging from cardiogenic to neurally mediated. While most of the syncopal episodes in the literature following mRNA vaccination are attributed to anxiety-related events, this was not the case in our patient. The World Health Organization mentions this to be a serious neurologic adverse event warranting reporting to VAERS despite not having been done by the patient's physicians previously [16]. A cardiogenic inflammatory response might have caused transient neurologic symptoms if myocarditis ensued, which propagated a transient arrhythmia in our 38-year-old patient, given the presence of his syncopal episode. Given that the relative risk of myocarditis in young males has shown to be higher after COVID-19 mRNA second vaccination vs. from infection, this is very troublesome given how many people are infected on top of being vaccinated and a true concern for many eligible children and adolescents [7]. Our case contributes to prior concerns of adverse events showing up only after already passing through authorization phase trials, which are defined as serious. While the pathology and location of S-spike protein deposition was also not an endpoint in any of the initial trials, we do know now that this has been shown to induce central nervous system (CNS) inflammation [17]. 

These three cases in young individuals with serious neurologic sequelae may, in fact, indicate only the beginning of adverse reactions lurking in younger populations as time and proper reporting are the only way to tell in the future. This raises concern of risk vs. benefit as there is no long-term safety with rising concerns in the short run. Meanwhile, having had a COVID-19 infection before vaccination also appears to raise the associated greater odds of adverse events, so this should absolutely be a consideration for exemption [18]. Individualized risk factors such as vitamin D deficiency, for example, are hardly mentioned and even ignored by the CDC, while a meta-analysis review shows that there may be a theoretical risk of zero for COVID-19 if vitamin D levels are optimized according to research [19,20]. As one-size-fits-all medicine may potentially be harming certain demographics, the advent of clinical genomics and integrative medicine to personalize care and decision-making may help influence public health strategies. This consideration should be a part of the recommendations by weighing risk vs. benefit more stringently.

## Conclusions

Despite the CDC's stance that neurological side effects following COVID-19 mRNA vaccines are rare and often mild, we see rising concerns for those 40 years old and under who remain at low risk from infection. With so many younger people already infected with acquired immunity, change needs to occur within our health agencies to portray a realistic risk of infection vs. immunization as coronavirus boosters become the norm. This is of grave concern to those under forty years of age, especially our youth, and should be more publicly addressed. Given post-authorization reporting of cardiac issues as well as neurological ones, it is crucial to note these in pre-authorization trials. As we have very little knowledge of what are the long-term issues, we need to consider the question of when do we implement benefit vs. risk evaluation to avoid unnecessary harm? The CDC must be more attentive to all the adverse events encountered as a result of these vaccines in the younger population. This is particularly concerning, as illustrated by our cases over such a short time window after vaccination. It is difficult to imagine the future side effects for those under seventeen years of age now being required to vaccinate despite herd immunity and being at minimal risk. These pressures are based on CDC guidance. Physicians and parents should also be more active in weighing risks vs. benefits by mandating informed consent and choice instead of one-size-fits-all practices. Hopefully, more studies will help shape the medical ethics around experimental vaccines in the young in the very near future to prevent unnecessary harm. 

## References

[1] Dong Y, Mo X, Hu Y, Qi X, Jiang F, Jiang Z, Tong S (2020). Epidemiology of COVID-19 among children in China. Pediatrics.

[2] Patone M, Handunnetthi L, Saatci D (2021). Neurological complications after first dose of COVID-19 vaccines and SARS-CoV-2 infection. Nat Med.

[3] (2021). Guillain-Barré syndrome and vaccines. https://www.cdc.gov/vaccinesafety/concerns/guillain-barre-syndrome.html.

[4] Baker MA, Kaelber DC, Bar-Shain DS (2015). Advanced clinical decision support for vaccine adverse event detection and reporting. Clin Infect Dis.

[5] Rosenblum HG, Hadler SC, Moulia D (2022). Use of COVID-19 vaccines after reports of adverse events among adult recipients of Janssen (Johnson & Johnson) and mRNA COVID-19 vaccines (Pfizer-BioNTech and Moderna): update from the Advisory Committee on Immunization Practices — United States, July 2021. Morb Mortal Wkly Rep.

[6] (2021). Table 2 Demographic characteristics of patients who had the individual outcomes in the 28 days following a COVID-19 vaccine first dose or SARS-CoV-2 infection in the vaccinated population in England from 1 December 2020 to 31 May 2021. https://www.nature.com/articles/s41591-021-01556-7/tables/2.

[7] Patone M, Mei XW, Handunnetthi L (2022). Risks of myocarditis, pericarditis, and cardiac arrhythmias associated with COVID-19 vaccination or SARS-CoV-2 infection. Nat Med.

[8] Hause AM, Baggs J, Marquez P (2021). COVID-19 vaccine safety in children aged 5-11 years — United States, November 3-December 19, 2021. Morb Mortal Wkly Rep.

[9] Theoharides TC, Conti P (2021). Be aware of SARS-CoV-2 spike protein: there is more than meets the eye. J Biol Regul Homeost Agents.

[10] Lechtenberg R, Shulman A (1984). The neurologic implications of tinnitus. Arch Neurol.

[11] Besedovsky HO, del Rey A (2011). Central and peripheral cytokines mediate immune-brain connectivity. Neurochem Res.

[12] Parrino D, Frosolini A, Gallo C, De Siati RD, Spinato G, de Filippis C (2021). Tinnitus following COVID-19 vaccination: report of three cases. Int J Audiol.

[13] Petruk G, Puthia M, Petrlova J (2020). SARS-CoV-2 spike protein binds to bacterial lipopolysaccharide and boosts proinflammatory activity. J Mol Cell Biol.

[14] Buzhdygan TP, DeOre BJ, Baldwin-Leclair A (2020). The SARS-CoV-2 spike protein alters barrier function in 2D static and 3D microfluidic in-vitro models of the human blood-brain barrier. Neurobiol Dis.

[15] La Mantia L, Erbetta A (2004). Headache and inflammatory disorders of the central nervous system. Neurol Sci.

[16] Garg RK, Paliwal VK (2022). Spectrum of neurological complications following COVID-19 vaccination. Neurol Sci.

[17] Khan S, Shafiei MS, Longoria C, Schoggins J, Savani RC, Zaki H (2021). SARS-CoV-2 spike protein induces inflammation via TLR2-dependent activation of the NF-κB pathway. eLife.

[18] Beatty AL, Peyser ND, Butcher XE (2022). Analysis of COVID-19 vaccine type and adverse effects following vaccination. JAMA Netw Open.

[19] Chambers Chambers, PW PW (2022). COVID- 19: from cough to coffin. Open Access Library Journal.

[20] Borsche L, Glauner B, von Mendel J (2021). COVID-19 mortality risk correlates inversely with vitamin D3 status, and a mortality rate close to zero could theoretically be achieved at 50 ng/mL 25(OH)D3: results of a systematic review and meta-analysis. Nutrients.

